# Strong, but Wrong: Lay People’s and Police Officers’ Beliefs about Verbal and Nonverbal Cues to Deception

**DOI:** 10.1371/journal.pone.0156615

**Published:** 2016-06-03

**Authors:** Glynis Bogaard, Ewout H. Meijer, Aldert Vrij, Harald Merckelbach

**Affiliations:** 1 Maastricht University, Department of Clinical Psychological Science, Section Forensic Psychology, Maastricht, The Netherlands; 2 University of Portsmouth, Department of Psychology, Portsmouth, The United Kingdom; Kyoto University, JAPAN

## Abstract

The present study investigated the beliefs of students and police officers about cues to deception. A total of 95 police officers and 104 undergraduate students filled out a questionnaire addressing beliefs about cues to deception. Twenty-eight verbal cues were included in the questionnaire, all extracted from verbal credibility assessment tools (i.e., CBCA, RM, and SCAN). We investigated to what extent beliefs about nonverbal and verbal cues of deception differed between lay people (students) and police officers, and whether these beliefs were in agreement with objective cues known from research. Both students and police officers believed the usual stereotypical, but non-diagnostic (nonverbal) cues such as gaze aversion and increased movement to be indicative of deception. Yet, participants were less inclined to overestimate the relationship between verbal cues and deception and their beliefs fitted better with what we know from research. The implications of these findings for practice are discussed.

## Introduction

Early research suggests we tell on average two lies each day [1]. More recent studies, however, have shown that there are large individual differences in the prevalence of lie telling, with the majority of lies being told by a minority of people [2–4]. All these studies suggest that everyone has experience with either being lied to, or with telling lies themselves. Yet, despite this personal experience with deception, research has shown that people, including trained police officers, only perform around chance level in detecting deception [5–8].

One possible explanation for the failure to detect deceit is that people often hold incorrect beliefs about which cues are diagnostic of deception. One notable example here is the belief that liars display more gaze aversion. The Global Deception Research Team [9] investigated the most widespread beliefs about cues indicative of deception, sampling 2320 participants from 58 countries. Over eleven thousand responses were obtained, resulting in 103 distinct beliefs. Gaze aversion was the belief mentioned by most (64%) participants. Comparable results have been obtained by Strömwall and Granhag [10], who reported that gaze aversion and an increase in body movement were believed to be strong cues of deception among police officers, judges, and prosecutors. Research shows, however, that gaze aversion is not a sign of deceit [11, 12].

Incorrect beliefs about cues to deception are not confined to gaze aversion. People tend to rely heavily on nonverbal cues when making deception verdicts (for more information see [13]), regardless of a large body of research showing that deception cannot be reliably inferred from behavior [11, 12]. Studies from the UK, The Netherlands and Sweden have compared professionals and lay persons’ beliefs about cues to deception, including various professions such as police officers, judges, customs officers, prison guards, and immigration officers as professional lie catchers. These studies revealed that professionals typically hold as many (nonverbal) incorrect beliefs about deception as lay people [10, 13–17]. Moreover, when tested against the deception literature, both professionals and lay people overestimated the number of cues that are actually associated with deception [18, 19]. More recently, Masip and Herrero [20] asked police officers and community members how lies can be detected. Again both groups primarily mentioned beliefs about nonverbal cues.

As people tend to rely primarily on nonverbal cues, the verbal content of the message is largely ignored, despite research showing that diagnostic accuracy can be improved when relying on content [21–24]. Additionally, Mann, Vrij [25] reported that good lie detectors relied more on verbal cues, while poor lie detectors relied more on nonverbal cues. Moreover, meta-analytic research reported a higher lie detection accuracy if the training was based on verbal cues compared with nonverbal training [21]. Consequently, content should accordingly be favored over behavior [20, 24, 26]. Surprisingly little research has, however, looked at beliefs about such content cues.

Several veracity assessment methods have been developed that rely specifically on the content of a statement, such as Criteria-Based Content Analysis (CBCA) [27] and Reality Monitoring (RM) [28]. CBCA is originally based upon the ‘Reality Criteria’ that were formulated by Undeutsch [29], but subsequently transformed by Steller and Köhnken [27] into the method as it is used to date. The CBCA consists of a list of 19 content criteria that are expected to be more present in true compared to fabricated statements. There is indeed evidence that liars generally tell a less coherent story and are less likely to make spontaneous corrections to their story (e.g., It was about 2 p.m., oh wait, no it was about 4 p.m.”). Also, liars describe fewer reproductions of conversations (e.g., He told me: “take off your pants, or someone will get hurt”). Typically, they will include more contradictions and tell their stories in a more chronological order, for example, because they tend to stick to their rehearsed story. Liars are also less likely to admit forgetting certain details about the event (e.g., “I know he was wearing a dark blue sweater, but I don’t remember the color of his pants”) [11, 23, 30, 31]. CBCA was originally developed for evaluating children’ s testimonies in cases of alleged sexual abuse, but several studies have shown that CBCA can also be used for adults, and is not restricted to sexual abuse cases [32–35]. Both field studies [36–38] and lab studies [34, 39–41] reported that CBCA is able to accurately discriminate between truthful and fabricated statements A qualitative review by Vrij [23], and more recently by Amado et al. [30] showed that the average accuracy rate of CBCA varies around 70%.

RM [28] originally stems from memory research and was initially used for evaluating whether a memory originated from a real experience or an imagined event. The rationale is that a memory from a real experience arises from perception and accordingly will contain more sensory, contextual, and affective information than memories that originate from imagination. It is also assumed that memories of real experienced events are more vivid, clear, and sharp than fabricated memories, which are usually more vague, less concrete, and are more likely to contain cognitive operations [33, 42]. Scholars have investigated the usefulness of RM as an aid in assessing credibility. A set of RM criteria has been proposed by Sporer [33] and entails criteria about specific types of details and items on the realism and clarity of the statement. Support has been found for a number of RM criteria, namely that liars include less perceptual (e.g., smell, taste, sound), spatial (e.g., location) and temporal (e.g., time, duration) information and that the stories of liars are less plausible than truth tellers’ stories [11, 43]. Meta-analytic reviews have shown that the overall accuracy is similar to that of CBCA and varies around 70% [43, 44]. Although both CBCA and RM have a considerable error margin, it is better than the alternative of relying on intuition (i.e., chance level, see [6, 7]).

As said, little research, however, has looked at people’s beliefs about the CBCA and RM cues. One example is Akehurst, Köhnken [14], where police officers’ and laypersons’ beliefs about 47 nonverbal cues and 17 content related cues were examined, the latter extracted from RM and CBCA (e.g., spatial and temporal information, emotions, description of conversations). However, the main focus of Akehurst et al. [14] was on investigating people’s beliefs about their own and other peoples’ deceptive behavior. Therefore, their study does not allow testing the accuracy of participants’ beliefs about verbal and nonverbal cues, and how lay people and police may differ in this respect. Vrij, Akehurst [16] used the same list of cues and asked police officers, social workers, teachers and the general public about cues to deception, and how these cues might differ depending on the age of the messenger. No differences between groups regarding their beliefs were reported. Again, the focus of the article was not on the accuracy of the separate cues, but on the group and age differences. Recent research has additionally shown that although both police officers and community members report mostly nonverbal cues when asked how lies could be detected, police officers mentioned more verbal cues [20].

The present study aimed to replicate and extend the previous findings of police officers’ and lay peoples’ beliefs about lie detection cues. In contrast to Akehurst et al. [14] and Vrij, Akehurst [16], we also explored participants’ views about verbal and nonverbal cues via an open-ended question. In this way, participants were permitted an unlimited number of possible answers, were able to clarify their responses, and could mention cues that were not anticipated on the basis of previous literature. This provides us with detailed information about which cues our participants associate with deception, without influencing them in any way. As stated above, people tend to focus on (invalid) nonverbal cues, but little is known about their insight in verbal cues. Therefore, we examined their views about verbal cues further by asking closed-ended questions related to 28 content cues, instead of 17 content cues used in the previously mentioned studies. These content cues were extracted from CBCA and RM. Moreover, in contrast to previous research, this study focused on the correctness of these beliefs. Insight in these cues is helpful as they shed light on how well practitioners are informed about deception research, and about verbal cues in particular. Preferably, their knowledge on the surveyed cues is better than those of undergraduates.

Besides the beliefs about CBCA and RM criteria, we were also interested in the beliefs about criteria derived from Scientific Content Analysis (SCAN). SCAN is a verbal credibility analyses tool, that has been developed by former polygraph examiner Sapir [45]. On the basis of his experience as a polygraph examiner he argued that truth tellers and liars differ in their language. Based on these assumed differences, Sapir developed criteria that could be used to identify deception. According to Sapir, his method is widely used in countries around the world (e.g., Australia, Belgium, Canada, Israel, Mexico, UK, US, the Netherlands, Qatar, Singapore, and South Africa), and is also used by Federal agencies, Military law enforcement, private corporations, and social services (retrieved from www.lsiscan.com/id29.htm). For example, the SCAN course is given on an annual basis to Belgian and Dutch police officers [46, 47]. Moreover, Vrij [31] describes that SCAN was known by most attendees of an international investigative interviewing seminar, and that many practitioners reported to apply it as a lie detection tool.

Despite its widespread use, no research has supported claims of SCAN’s diagnostic accuracy and several studies showed that the SCAN criteria could not differentiate between true and fabricated accounts [39, 40, 48–50]. In a previous study, we investigated whether–in absence of diagnostic accuracy–susceptibility to confirmation bias could serve as an alternative explanation for SCAN’s popularity [40]. In the current study, we extend this line of research by including SCAN criteria to investigate whether SCAN’s popularity could be explained by the intuitive plausibility of its items. More precisely, we were interested whether the content criteria used in SCAN would fit with the beliefs people hold about these criteria.

To sum up, the current study explored three issues. First, we investigated which beliefs undergraduates and police officers hold about lie detection in general via an open-ended question, and whether these beliefs were in accordance with the deception literature. Second, we explored the specific beliefs of both groups regarding the 28 content cues via specific questions, and again checked these beliefs against the deception literature. Third, to test whether beliefs can account for the popularity of SCAN, we investigated to what extent the beliefs about SCAN criteria of both groups were in agreement with the hypothesized direction. Given the exploratory nature of our research, we have not formed specific hypotheses.

## Method

### Participants

A total of 199 participants filled out a questionnaire containing items about cues to deception (see below). The sample consisted of 95 police officers (*M*_age_ = 44, 64 men) and 104 undergraduate students (*M*_age_ = 19, 18 men). The police officers were recruited by approaching as many police stations as possible, both by phone and by mail informing them about our research. Police officers who expressed an interest in participating were asked to contact the experimenters and were send the link to the questionnaire via email. Participants came from different cities across the Netherlands (e.g., Almelo, Deventer, Groningen, Assen, Maastricht, Sittard, Roermond, Eindhoven, Utrecht, Den Haag). Police officers were either detectives or professional interrogators, so they all had experience with conducting interrogations. They reported a mean experience of 22 years (*SD* = 9.72) ranging from 2.5 to 40 years.

The undergraduate students were recruited through flyers and advertisements at our university campus, or via an online participation system of our university. Participants had an average age of 19 years (*SD* = 1.52) and were mainly first and second year psychology students. These students were included, as they had not yet received any information on lie detection or interviewing techniques in their curriculum. Undergraduates received credit points, whereas the police officers did not receive any compensation for their participation. The study was approved by the ethical committee of the Faculty of Psychology and Neuroscience of Maastricht University. Participants read and signed the appropriate informed consent in accordance with the Declaration of Helsinki and were guaranteed that they could resign from the project at any time and without any consequences.

### The questionnaire

Participants first read and signed the online version of the informed consent before starting with the questionnaire. After signing the informed consent, they were asked the open-ended question: “What do you think are good cues for detecting lies?” Participants were given unlimited space to respond. Next, they were asked to indicate their opinion about 28 content cues. Twenty-six of these cues were derived from known verbal credibility assessment tools, namely CBCA [27], RM [28], and SCAN [45].

For CBCA, we included 13 items and excluded the items that have shown to be only rarely present in statements and received little empirical support (i.e., related external associations, raising doubts about one’s own testimony, self-deprecation, pardoning the perpetrator and details characteristic of the offence) [23, 30]. Furthermore, we excluded the item a*ccurately reported details misunderstood* as it is used primarily for evaluating child statements. For RM, we included all eight items described by Sporer [33]. The list of SCAN is very elaborate, with some sources reporting as many as 28 items (see[51]). For the current study we included only those criteria that have been shown to be most frequently used in practice [51]. This resulted in a list of 12 criteria that are also reported in Vrij [31]. Some of the criteria included in the SCAN are thought to appear more often in deceptive statements, while others are believed to appear more often in truthful statements. As there was CBCA, RM, and SCAN overlap with regard to six items (i.e., spontaneous corrections, lack of memory, emotions, spatial information, temporal information, extraneous information), we included these items only once. Additionally, we included the item “length” in our survey, as research has shown that truthful stories tend to be longer than fabricated ones [11]. Moreover, we have included the item “self-references”, which is a combination of two SCAN criteria (i.e., use of pronouns and first person singular, past tense), and has been shown to be diagnostic in previous studies (see for example [11, 52]). This resulted in a list of 28 separate items, which were presented in the order listed in Appendix A.

For every item we gave a short description illustrated by an example. For example, for spatial information we gave the following description “This cue refers to all descriptions about locations or spatial arrangements of people and/or objects (e.g., He was sitting left to his wife)”. Next, as in Strömwall and Granhag [10] and Granhag et al. [53] participants were asked to indicate their opinion on forced-choice answer scales with four alternatives, for all items. Respondents could choose between two directed (e.g., ‘this cue is used less by liars’ or ‘this cue is used more by liars’) and one neutral (e.g., there is no difference between liars and truth tellers regarding this cue’). Furthermore, a ‘don’t know’ alternative was also always available.

Additionally, participants answered questions about their background, function and experience, and whether they ever had training in deception detection. Furthermore, participants were asked to indicate on a 7-point scale ranging from 1 (very poor) to 7 (very good) how well they thought they would perform in detecting deception and how well they knew the literature about lie detection. The questionnaire was administered online via thesistools.com and all participants received a login name and code to complete the questionnaire. To make sure that the participants completed the entire questionnaire, it was built in such way that participants could not skip any questions. Two students were asked to pilot the questionnaire; they needed approximately 30–45 minutes to complete all items.

## Results

None of the participants reported ever having received training in lie detection. In the following we present the answers both groups gave to our questions about their understanding of lie detection literature and their skills in detecting deceit. First, in response to the question ‘how well do you know the literature about lie detection?’, the police officers reported they were not very knowledgeable about the literature (*M* = 2.78, *SD* = 1.47), but still, police officers’ self-reported knowledge was more extensive than that of students (*M* = 1.89, *SD* = 1.03) [*t*(197) = 4.94, *p* < 0.001, *d* = 0.71]. In response to the question ‘how well do you think you would perform in detecting deception?’, police officers indicated their self perceived performance to be moderate (*M* = 3.92, *SD* = 1.31), which did not differ from the students (*M* = 4.15, *SD* = 0.95) [*t*(197) = 1.41, *p* = 0.16, *d* = -0.20]. Students who indicated they were more knowledgeable about the literature also indicated they were better at detecting deceit (*r*(105) = .332, *p* = 0.001). In contrast, no significant correlation between literature knowledge and deception detection was found for police officers. However, we did find that experience as an officer (in years) was positively correlated to self-reported lie detection skills (*r*(95) = .213, *p* = 0.038).

We first present the results for the open question using an analysis similar to the one reported by The Global Deception Research Team [9]. Next, we present the results for the closed questions using an analysis similar to that of Strömwall and Granhag [10] who used a similar response format. In the following sections the analyses will be clarified in more detail.

### Open question

In response to the question: “What do you think are good cues for detecting lies?” widely different responses were obtained. To condensate the data, two raters examined all responses and grouped them into two different categories; nonverbal and verbal cues. Within the nonverbal category, responses were assigned to specific categories such as speech characteristic (e.g., response latency, voice pitch), facial behaviors (e.g., blushing, gaze aversion), and body movements (e.g., illustrators, moving feet). For this purpose, the list of 47 categories employed by Akehurst and colleagues for the complete list see [14, 16] was used. The verbal cues were categorized according to the cues listed in Appendix A.

First, inter-rater reliability of the two raters for presence of cues in responses of participants was calculated. We only coded a cue as present when both raters agreed upon its presence, when raters disagreed upon its present, the cue was scored as absent. As can be seen in Tables 1 and 2, percentages often deviated considerably from 50%, which indicates a skewed data set. This is potentially problematic as Kappa is not an informative measure of agreement with highly skewed marginal distributions. In such cases, the reported Kappa value can in fact be very misleading (see for instance [54]). To overcome the misleading underestimation of Kappa in our dataset, we also included percentage agreement. As can be seen in Tables 1 and 2, for nearly all cues the prevalence is very low, which explains the discrepancy between percentage of observed agreement (high) and the chance corrected agreement of the Kappa statistic (low). As the low Kappa values were always accommodated by high levels of observed agreement, our values can be considered sufficient to continue our analyses.

**Table 1:** Percentage of students (n = 104) and police officers (n = 95) mentioning nonverbal cues.

	Students			Police officers		
Items	Percentage Present	Kappa	Percentage agreement	Percentage Present	Kappa	Percentage agreement
Gaze aversion	51.9	0.88	94.2	23.2	0.92	97.9
Nervous body	35.6	0.86	93.3	10.5	0.85	96.8
Sweating	27.9	0.98	99	11.6	0.95	98.9
Body movements	15.4	0.9	97.1	8.4	0.69	93.7
Facial expressions	10.6	0.82	96.2	5.3	0.9	96.8
Blushing	8.7	1	100	10.5	-	100
Stuttering	7.7	0.88	98.1	7.4	0.93	98.9
Self manipulations	6.7	0.81	97.1	3.2	0.85	98.9
Faltering speech	5.8	0.79	97.1	1.1	0.49	100
Pitch	5.8	0.85	98.1	2.1	1	100
Hand arm finger movements	5.8	0.76	97.1	3.2	0.85	98.9
Repetitions	4.8	0.9	99	2.1	-	97.9
Postural shifts	4.8	0.76	97.1	10.5	0.81	95.8
Speech characteristics	3.8	0.79	98.1	2.1	1	100
Hectic speech	2.9	1	100	1.1	1	97.9
Pupil dilation	2.9	1	100	0	-	100
Smiling	2.9	1	100	0	-	100
Gesticulations	2.9	0.65	97.1	0	-	100
Behavior (not further specified)	1.9	0.8	99	27.4	0.8	91.6
Evasive responses	1.9	1	100	5.3	0.64	94.7
Response latency	1.9	0.8	99	4.2	0.88	98.9
Eye blinks	1.9	1	100	0	-	100
Shaking	1.9	1	100	3.2	1	100
Leg feet movements	1.9	1	100	3.2	1	100
Grammatical errors	1	0.66	99	1.1	1	100
Soft voice	1	1	100	0	-	100
Swallowing	1	1	100	0	-	100
Head movements	1	1	100	0	-	100
Nervous face	1	0.66	99	0	-	98.9
Pauses	0	-	100	1.1	1	100
Twitches	0	-	100	2.1	0.66	100
Variation in facial	0	-	99	1.1	-	98.9
Change in behavior	0	-0.1	98.1	6.3	0.73	95.8

*Note*. Although we used the 48 items presented in Akehurst et al. [14] to categorize the answers of our participants, only 33 different items of this list were actually covered within the answers.

**Table 2:** Percentage of students (n = 104) and police officers (n = 95) mentioning verbal cues.

	Students			Police officers		
Items	Percentage Present	Kappa	Percentage agreement	Percentage Present	Kappa	Percentage agreement
Contradictions	8.7	0.94	99	31.6	0.86	93.7
Quantity of details	10.6	0.83	96.2	3.2	0.74	97.9
Verbal	0	-	98.1	3.2	1	100
Coherence	0	-	99	2.1	1	100
Plausibility	0	-	99	2.1	1	100
Lack of memory	0	-0.1	98.1	1.1	0.49	97.9
Missing information	0	-	100	1.1	1	100

*Note*. Although we used the 28 items of Appendix A to categorize the answers of our participants, only 7 different items of this list were actually covered within the answers of our respondents.

To our question “What do you think are good cues for detecting lies?”, participants gave a total of 443 different responses. For students, the two raters identified 232 nonverbal cues and 20 verbal cues; for police officers, these were 149 nonverbal cues and 42 verbal cues. Thus, on average, 14 percent of the total responses pertained to verbal cues of deception. Looking at the distinct categories displayed in Table 1, the most common nonverbal cues about deception mentioned by students were (1) gaze aversion, (2) nervousness, (3) sweating, (4) body movements, and (5) facial expressions (not further specified). For police officers the most common nonverbal cues were (1) behavior (not further specified), (2) gaze aversion, (3) sweating, (4) nervousness, and (5) blushing. Chi-square analyses were used to identify significant differences between groups. For both students and police officers, *gaze aversion* was the most frequently mentioned cue, but students mentioned it more often than police officers, [χ^2^ (1, N = 199) = 17.40, *p* < 0.001]). For the remaining cues, the cue *behavior* was mentioned more often by police officers than by students [χ^2^ (1, N = 199) = 26.59, *p* < 0.001]), while students mentioned *sweating* [χ^2^ (1, N = 199) = 8.22, *p* = 0.004]), and *nervousness* [χ^2^ (1, N = 199) = 17.27, *p* < 0.001]) more often than police officers. For *facial expressions*, *blushing* and *body movements*, no significant differences emerged between groups.

For both students and police officers, the most common verbal cues were (1) contradictions and (2) quantity of details. Students mentioned *quantity of details* more often than police officers [χ^2^ (1, N = 199) = 4.18, *p* = 0.04]), while police officers mentioned *contradictions* more often than students [χ^2^ (1, N = 199) = 16.56, *p* < 0.001]). Some police officers (3.2%) said they used verbal cues to detect deception but did not specify these cues. Thus, police officers and students listed considerably more (four and 11 times, respectively) nonverbal cues than verbal cues as diagnostic cues.

### Closed questions

#### ‘Don’t know’ answers

Table 3 summarized endorsement percentages in students and police officers. We first investigated to what extent both groups chose the ‘don’t know’ alternative. To allow for Chi-square tests, we first recoded the data such that both directed answers (i.e., less or more) and the neutral answer (i.e., no difference) were coded as ‘1’ and the ‘don’t know’ answer was coded as ‘0’. Next, we compared the two groups with each other: 27 out of 28 Chi-squares were significant, meaning that for all but one item (i.e., plausibility [χ^2^ (1, N = 199) = 2.51, *p* = 0.11]), police officers were more conservative, and chose the ‘don’t know’ answer significantly more often than student (i.e., Chi-square values ranged between 6.13 and 19.78).

**Table 3:** Percentage of students (n = 104) and police officers (n = 95) who endorsed the answer options.

	Students				Police officers			
Item	Negative	No difference	Positive	Don't know	Negative	No difference	Positive	Don't know
Denial of allegation	17.31	10.58	64.42	7.69	11.58	27.37	29.47	31.58
Social introduction	34.62	18.27	35.58	11.54	27.37	21.05	15.79	35.79
Coherence	67.31	8.65	24.04	.00	41.05	26.32	18.95	13.68
Clarity	44.23	22.12	31.73	1.92	34.74	20.00	23.16	22.11
Spontaneous corrections	47.12	9.62	38.46	4.81	26.32	15.79	35.79	22.11
Lack of memory	42.31	17.31	37.50	2.88	22.11	14.74	45.26	17.89
Contradictions	12.50	10.58	74.04	2.88	14.74	15.79	51.58	17.89
Perceptual information	62.50	15.38	12.50	9.62	60.00	10.53	6.32	23.16
Main event of statement	42.31	10.58	32.69	14.42	49.47	3.16	20.00	27.37
Emotions	60.58	13.46	18.27	7.69	49.47	17.89	7.37	25.26
Quantity of details	40.38	11.54	43.27	4.80	54.74	12.63	15.79	16.84
Spatial information	46.15	22.12	29.81	1.92	50.53	15.79	11.58	22.11
Objective versus subjective time	50.96	11.54	24.04	13.46	36.84	13.68	8.42	41.05
Unstructured production	31.73	21.15	40.38	6.73	25.26	24.21	31.58	18.95
Description of interaction	51.92	19.23	19.23	9.65	45.26	11.58	12.63	30.53
Temporal information	31.73	29.81	32.69	5.81	41.05	21.05	12.63	25.26
Self-references	38.46	14.42	40.38	6.73	33.68	16.84	20.00	29.47
Extraneous information	34.62	3.85	55.77	5.77	24.21	12.63	42.11	21.05
Missing information	34.62	17.31	42.31	5.77	22.11	15.79	34.74	27.37
Reproduction of conversation	57.69	14.42	19.23	8.65	51.58	10.53	7.37	30.53
Reconstructability	37.50	28.85	25.00	8.65	38.95	24.21	15.79	21.05
First person singular, past tense	31.73	28.85	16.35	23.08	20.00	17.89	6.32	55.79
Use of pronouns	21.15	37.50	25.96	15.38	16.84	18.95	12.63	51.58
Unusual details	63.46	8.65	23.08	4.81	53.68	11.58	12.63	22.11
Plausibility	37.50	36.54	18.27	7.69	46.32	28.42	10.53	14.74
Changes in language	39.42	12.50	38.46	9.62	14.70	20.00	22.10	43.20
Length of the statements	25.00	9.62	57.69	7.69	29.47	25.26	16.84	28.42
Cognitive operations	52.88	14.42	20.19	12.50	47.37	12.63	12.63	27.37
Average	41.30	17.10	33.60	7.90	35.30	17.40	20.00	27.30

*Note*. Negative means less or fewer when lying, positive indicates more when lying.

#### Directional and neutral answers

We analyzed the data as has been done previously in studies using a similar response format [10]. We first recoded directional and neutral answers as -1 (i.e., less when lying), 0 (i.e., no difference), and 1 (i.e., more when lying). We excluded the ‘don’t know’ alternative from the following analysis.

Next, we analyzed the data with multiple one-sample sign tests (one for every item in the questionnaire) to investigate whether the average mean value of every item was significantly different from 0. In this way we were able to investigate whether there was a preference for either one of the directional answers (i.e., more or less). Means and *p*-values for both groups are presented in Table 4. To correct for multiple testing, we have adjusted the alpha level to 0.01. Both groups believed that deceptive statements contained more *denials of allegations*, and more *contradictions* than truthful statements. On the other hand, both groups believed that deceptive statements were less *coherent*, contained less *perceptual information*, fewer *descriptions of emotions*, fewer *descriptions of interactions*, and fewer *reproductions of speech*. Moreover, both groups thought that for liars *objective and subjective time* were less in correspondence than for truth tellers.

**Table 4:** Beliefs about verbal cues of students (n = 104) and police officers (n = 95).

	Students			Police officers	Between groups	
Items	Mean	*p*-value	mean	*p*-value	*Z*-value	Correct answer^1^
Denial of allegation	.51	< .01	.26	.01	-2.58**	-
Social introduction	**.01**	**1.00**	**-.18**	**.12**	-1.30	-
Coherence	**-.43**	**< .01**	**-.26**	**< .01**	-1.90	<
Clarity	**-.13**	**-.17**	**-.15**	**.18**	-.13	-
Spontaneous corrections	-.09	.40	.12	.30	-1.50	<
Lack of memory	-.05	.66	.28	< .01	-2.43	<
Contradictions	**.63**	**< .01**	**.45**	**< .01**	1.84	>
Perceptual information	**-.55**	**< .01**	**-.70**	**< .01**	1.34	<
Main event of statement	**-.11**	**.31**	-.41	< .01	-2.01*	-
Emotions	-.46	< .01	-.56	< .01	-.51	-
Quantity of details	.03	.83	**-.47**	**< .01**	-3.56**	<
Spatial information	-.17	.07	**-.50**	**< .01**	2.58*	<
Objective versus subjective time	-.31	< .01	-.48	< .01	.95	-
Unstructured production	.09	.36	.08	.50	-.15	>
Description of interaction	-.36	< .01	-.47	< .01	-.91	-
Temporal information	.01	1.00	**-.38**	**< .01**	-3.04*	<
Self-references	.02	.91	-.19	.09	-1.47	<
Extraneous information	.22	.03	.23	.04	-.16	-
Missing information	**.08**	**.43**	**.17**	**.13**	-.63	-
Reproduction of conversation	**-.42**	**< .01**	**-.64**	**< .01**	-1.64	<
Reconstructability	**-.14**	**.14**	-.29	< .01	-1.24	-
First person singular, past tense	-.20	.03	-.31	.02	-.70	-
Use of pronouns	.06	.57	-.09	.57	-1.04	<
Unusual details	-.42	< .01	-.53	< .01	-.60	-
Plausibility	**-.21**	**.01**	**-.42**	**< .01**	-1.92	<
Changes in language	**-.01**	**1.00**	**.13**	**.31**	-.90	-
Length of the statements	.35	< .01	-.18	.10	-4.00**	<
Cognitive operations	-.37	< .01	-.48	< .01	-.74	-

*Note*. Minus sign indicates that the specific criterion is less present for liars.

> Verbal characteristics occurs more frequently in deceptive statements

'- No relationship between the verbal characteristic and lying/truth telling

< Verbal characteristics occurs less frequently in deceptive statements.

*p<0.01.

**p<0.001. Beliefs in the correct direction are in bold.

^1^According to Depaulo et al. [11], Masip et al. [43], Nahari et al.[48], Newman and Pennebaker [52] and Vrij [23, 31].

Students and police officers also believed that liars use less *first person singular past tense* verbs; tell stories that are less *plausible*, with fewer *unusual details*, and fewer *cognitive operations*. Both groups believed that there was no difference between truth tellers and liars concerning the following cues: *Social introduction*, *clarity*, *spontaneous corrections*, *unstructured production*, *self-references*, *extraneous information*, *missing information*, *use of pronouns*, and *changes in language*.

We also investigated whether police officers and students significantly differed in their preference for the items in the questionnaires. This was done by means of multiple Mann-Whitney tests (for every item separately). Again, to correct for multiple testing, we have adjusted the alpha level to 0.01. Although both groups were in agreement for most of the cues, they significantly differed in their opinion on six cues (see Table 4). Students expressed the belief that deceptive statements were *longer* than truthful statements, while police officers thought there was no difference in this respect. For the remaining five cues (i.e., *main event of the statement*, *quantity of details*, *temporal and spatial details*, *and reconstructability of the statement*), police officers thought they were less present in deceptive statements, while students believed there were no differences between deceptive and truthful statements with regard to these five cues.

#### Relationship with extant literature

Table 4 summarizes which verbal cues are solid cues of deception according to the extant empirical literature. If evidence about a particular item was mixed, the item was denoted with a -, indicating no clear relationship between the verbal characteristic and deception. Items that were exclusively derived from SCAN (i.e., 8 items, see Appendix A) are discussed in the next section.

As is clear from Table 4, not all items in our questionnaire are shown to be effective when detecting lies. This section gives a detailed overview of how we derived to the decision of diagnosticity. RM items were scored following the results of DePaulo, Lindsay [11] and Masip, Sporer, Gardio, and Herrero [43]. A RM item was scored as diagnostic if the item was significantly more present in truthful statements compared to fabricated statements (or vice versa for cognitive operations) in more than 65% of the included studies. CBCA items were scored on the basis of Amado, Arce [30], DePaulo et al. [11] and Vrij [23, 31]. A CBCA item was scored as diagnostic if the item was significantly more present in truthful statements compared to fabricated statements in more than 64% of the studies included in the meta-analyses of Vrij [23, 31] and/or showed an effect size of at least *d* = 0.50 in Amado, Arce [30], and/or a significant difference in DePaulo, Lindsay [11]. The remaining items—use of self-references and length of statement–were evaluated on the basis of Newman and Pennebaker [52] and DePaulo et al. [11] and were scored as diagnostic as both reported significant differences between truthful and fabricated statements regarding these items.

**Evaluation of diagnostic cues -** Five out of these 13 diagnostic cues were correctly judged by both students and police officers, namely *coherence*, *contradictions*, *perceptual information*, *reproduction of conversation*, *and plausibility*. Police officers additionally judged *quantity of details* and *spatial* and *temporal information* correctly (Table 5). In sum, as a group, students evaluated five out of 13 diagnostic cues (38%) correctly, while police officers judged eight out of 13 cues (62%) correctly.

**Table 5:** Detailed overview of the empirical merits of the beliefs of students (n = 104) and police officers (n = 95).

	Number of items	
Possible outcomes	Students	Police officers
*Diagnostic cues (13 items)*		
Correct	5 (38%)	8 (62%)
Incorrect	8 (62%)	5 (38%)
*Non-diagnostic cues (7 items)*		
Correct	2 (29%)	1 (14%)
As hypothesized	3 (42%)	4 (57%)
Incorrect	2 (29%)	2 (29%)
Total correct	7 (35%)	9 (45%)
Total correct and as hypothesized	10 (50%)	13 (65%)

For two cues, the groups held beliefs that ran counter to the empirical database; students believed liars gave *longer* statements and police officers thought liars were more likely to admit a *lack of memory*. For the remainder of these 13 diagnostic cues, both groups believed these criteria were not related to deception (i.e., seven for students, four for police officers), yet on the basis of deception literature, there is every reason to assume that they are.

**Evaluation of non-diagnostic cues -** This leaves us with seven cues that have no basis in empirical evidence, as can be seen in Table 4. For the items *clarity*, and *extraneous information* both groups correctly indicated truth tellers and liars did not differ regarding this item. Students also correctly judged *reconstructability* was not diagnostic to detect deceit. For all other items, both groups held the belief that these cues were diagnostic. We investigated to what extent these opinions were in agreement with the CBCA and RM hypotheses.

For three of them (i.e., emotions, descriptions of interactions, and unusual details), students and police officers indeed followed the hypothetical direction of the CBCA and RM instruments (i.e., criteria are less present when lying). Additionally, police officers’ belief about *reconstructability* also followed the hypothesis of RM (i.e., less when lying).

For two items both groups mistakenly believed liars included fewer *cognitive operations*, which is contrary to RM and CBCA’s hypotheses. Table 5 gives a detailed overview of the overall correctness of both groups.

Lastly, we compared the average beliefs about the diagnostic items to those of the non-diagnostic items. We recoded the scores for all the items: the correct answer was coded as 1, the incorrect answer(s) as 0. Next, we calculated the mean scores for the 13 diagnostic, and the seven non-diagnostic items. Two paired samples t-test on these means showed that beliefs about the diagnostic cues were more correct than about the non-diagnostic cues: students judged the diagnostic items (*M* = 0.40, *SD* = 0.17) higher than the non-diagnostic items (*M* = 0.16, *SD* = 0.18) [*t*(103) = 8.79, *p*< 0.001, *d* = 1.32], and so did police officers (*M* = 0.38, *SD* = 0.21 vs. *M* = 0.16, *SD* = 0.22) [*t*(94) = 6.68, *p*< 0.001, *d* = 1.03].

#### Relationship with SCAN hypotheses

According to the SCAN hypotheses, liars are less likely to directly deny the crime (e.g., they will try to divert from the topic), will fail to correctly introduce persons in their statements (e.g., a correct introduction includes name and role “my son Alex”) and will try to keep the description of the critical event as short as possible. Moreover, the objective and subjective time of their story will not correspond as well as with truth tellers, liars will have more information missing in their stories, use fewer pronouns and include more changes in language. Nahari et al. [48] reported no significant differences for any of the SCAN criteria, but Newman and Pennebaker [52] found evidence that liars included fewer pronouns in their statements. As a result, we know that all of the SCAN criteria, except for pronouns, lack diagnostic accuracy. Therefore, we were more interested to what extent the beliefs of both groups were in agreement with the hypothesized direction of SCAN. In this way, we were able to investigate how intuitively appealing the SCAN items are.

For two out of eight criteria—*objective and subjective time* and *first person singular past tense*—both groups agreed with the hypotheses of SCAN. Police officers additionally agreed with the hypothesis that liars describe the *main event* in less detail than truth tellers, while students believed there was no difference between liars and truth tellers in this respect. Note, that there is no empirical evidence to back up these criteria.

For four criteria—*social introduction*, *missing information*, *use of pronouns*, and *change in language*—both groups thought that there exists no difference between truth tellers and liars. For one criterion, *denial of allegations*, both groups believed that liars are more likely to deny the allegations, when in fact SCAN’s hypothesis states the opposite. The only item of the SCAN list that has been shown to be useful for detecting deception is *use of pronouns*; nonetheless, both groups believed it was not helpful. In total, students followed the hypothesized direction for two out of the eight SCAN items (25%) and police officers for three items (38%).

#### Correlational evidence for number of correct items

Finally, it was investigated whether the number of correctly judged items correlated with our participants’ self reported lie detection skills, knowledge on literature and years of experiences. For police officers years of experience (*r*(95) = .213, *p* = .038) and self reported lie detection skills (*r*(95) = .266, *p* = .009) positively correlated with the number of correct answers on the questionnaire. For students only their self reported knowledge on the literature positively correlated with the number of correct answers (*r*(95) = .214, *p* = .029).

## Discussion

The current study investigated the beliefs that students and police officers hold about deception. It expands on the extant literature by investigating an extensive list of verbal cues rather than focusing solely on nonverbal cues to deception. Three important issues were explored in this study.

When students and police officers were given the opportunity to list the cues they believed are indicative of deception, they predominantly listed the stereotypical, and unsupported, nonverbal cues (e.g., gaze aversion, nervousness, movement and sweating). These results are in agreement with previous findings [10, 13–17, 53, 55], but they also replicate more recent results [20]. In addition, both groups listed considerably more nonverbal than verbal cues as diagnostic cues. This is in line with studies showing that people tend to focus more on nonverbal cues than on verbal cues [10, 14, 16, 53], even though the latter are actually more diagnostic cues to deceit (for a review see [24]).

Two important differences emerged between police officers and students. First, in response to the open question, police officers reported overall less cues than students (191 vs. 252). Second, police officers mentioned more verbal cues than student (42 vs. 20 cues). This finding partially contradicts research by Masip and Herrero [20], who found that police officers overall reported more cues than lay people. Their data were derived from 22 Spanish officers who were asked to participate during a workshop on eyewitness psychology at their police department, and compared with the answers of 22 community members who were tested in public areas in the same town. Although a number of differences between policing in Spain and the Netherlands may account for this difference, one notable explanation could be that Dutch police officers are informed during their interrogation training to refrain from making credibility judgments based on behavioral signs [56]. As such they may be weary of nonverbal cues, and list fewer of them.

In terms of nonverbal cues of deception, our findings that especially gaze aversion, nervousness, movements and sweating were reported as cues to deception, fit with the mistakenly held assumption that liars are more anxious/nervous than truth tellers [57]. These cues are even reported outside the legal field. Within healthcare professions, nurses as well as therapists have also been shown to hold these false beliefs about deceptive cues [58, 59]. Moreover, Hart, Hudson, Fillmore and Griffith [60] compared managers and non-managers’ beliefs about deception cues on the work floor and also reported similar results. This incorrect, and widespread assumption, might be a result of the common view that lying is bad [9], and that liars should therefore feel afraid of getting caught. By this understanding, gaze aversion and increases in body movement signal the nervousness that liars feel about their moral dilemma. Actually, most of the behavioral cues that have been mentioned by our participants can be traced back to the idea that lying causes liars to feel distressed, and that this distress is shown in their facial expressions (i.e., blushing, sweating, blinking) or their body (movements, fidgeting, illustrators). However, people seem to underestimate the importance of situational factors that might influence someone’s behavior. For example, truth tellers can also be nervous for other reasons than deceptiveness, such as an accusatory interviewing style, the fear of not being believed, or the mere fact of being accused of a criminal act may result in nervous behavior [61].

The second part of our survey investigated beliefs about 28 specific content cues. Students and police officers were largely in agreement about the diagnosticity for most of the listed cues (i.e., 21 cues). For the directional questions, police officers more often chose the “don’t know” answer alternative than students (25% vs. 8%). This suggests that police officers adopt a more conservative threshold for cues to deceit than students. Although many reasons might account for this finding, the most likely explanation is that officers were more concerned with making mistakes than undergraduates. The last decade, much research has focused on investigating police practices, and many of those studies critiqued current practices (e.g., research on interrogation tactics and false confessions, see [62, 63]). Officers’ awareness that they were participating in scientific research possibly made them more hesitant to choose a directed response, minimizing their chances of making mistakes.

Interestingly, Strömwall and Granhag [10] reported a lower percentage of “I don’t know” answers for police officers (i.e., 10%) than the current study. One potential explanation for this difference is that we, unlike Strömwall and Granhag [10], tested participants’ beliefs on specific content cues. Participants may have simply been less familiar with these cues, resulting in an increased percentage of 'don't know' answers. Importantly, the relative high percentage of don’t know answers for police officers in our study means the results pertaining to the closed questions only reflect the participants who gave a directional answer. On average this amounts to 75% of the police officers, yet for some criteria (i.e., first person singular past tense and use of pronouns) this actually reflects less than half of the sample.

For the SCAN items, both groups reported beliefs in accordance to the hypothesized direction only for two items, meaning our data does not support our hypotheses that its intuitively appealing items explains SCAN’s popularity. Note that previous research failed to support SCAN’s diagnostic accuracy, so its popularity cannot be attributed to its accuracy [39, 40, 48, 51]. However, our results might be influenced by the way we presented the SCAN criteria. For every criterion included in our questionnaire we gave a description to explain the criterion and we provided participants with an example of one or two sentences. However, in the SCAN manual, the criteria and their interpretation are only vaguely described [45]. Research has shown inter-rater consistency to be low for SCAN in the field, suggesting practitioners adapt the criteria to their own needs [51, 64]. It might be precisely the lack of clear guidelines for the criteria that appeals to practitioners.

We also investigated whether beliefs about the verbal cues were in agreement with the empirical deception literature. Excluding the SCAN items, results revealed that both groups only showed an opposite belief (i.e., less vs. more) for three items. This means that for most of the items participants judged them correctly, or judged them as is hypothesized by CBCA and RM. This was confirmed by the analysis were the diagnostic and non-diagnostic cues were clustered. This finding may explain why untrained lie catchers relying on content cues tend to reach higher accuracy scores than do lie catchers relying on nonverbal cues [65–67].

Our results add to a body of evidence showing peoples’ beliefs in behavioral cues are not indicative of deception. But why are these incorrect beliefs so persistent? Besides reasons that are common in legal psychology, such as illusory correlations and confirmation bias [68–71], two other reasons are particularly important here. First, people—and especially police officers—usually receive inadequate or delayed feedback concerning their credibility judgments [57]. That is, for feedback to be effective, an officer should be informed about the truthfulness of the suspect directly after each interview. This does not happen in real life. However, adequate feedback is essential for learning, because people can adjust their decision-making strategy accordingly [72]. Indeed, lie detection training has been shown most efficient when the training combines information, practice with examples, and feedback [73]. Without feedback, people are not able to learn their nonverbal beliefs are generally wrong.

Second, police manuals often report subjective ideas on cues to deception instead of relying on scientific research [57]. Although this is particularly the case for manuals used in the US, many of the non-diagnostic cues included in these manuals have found their way into popular media (e.g., TV-series and movies). As a consequence, peoples opinions can be contaminated by the incorrect message that is conveyed about useful deception cues, as is the case for criminal profiling [74]. More precisely, people rely on anecdotal evidence showing that liars display these stereotypical behaviors. Also repetition of the message that deception can be detected by relying on stereotypical cues strengthens the illusion. Moreover, people often tend to accept information that is conversed to them by presumed experts. As such, if information on these cues can be found in police manuals, people will accept this as evidence for their accuracy.

Two important limitations of our study deserve some attention. First, as we made use of a questionnaire, we can never be sure that participants understood all the items that were included, or what their reasoning was for choosing a specific answer. By providing additional information and an example, we have tried to minimize this issue. Second, we only investigated beliefs and did not look at actual deception detection performance, leaving open the question whether participants with empirically grounded beliefs are actually better lie detectors [19]. Peoples’ self-reports about deception tactics do not always correspond with their actual decision making judgments. Nevertheless, there is vast literature showing that people are generally poor at lie detection [5, 6] and one reason for this is that valid cues to deception are rare. Importantly, even the best discriminating nonverbal cues correlate only modestly to deception [11, 18]. Furthermore, people have a tendency to strongly rely on nonverbal cues during deception detection [20]. Our finding that participants had less stereotypical beliefs about verbal cues to deception, might therefore explain the increased accuracy levels for lie detection when behavioral cues are actively excluded [67, 75, 76]. In any case, whether individuals who hold correct beliefs about verbal cues are actually better lie detectors is an issue that warrants future research.

In most interrogation settings, police officers have access to visual, vocal, and verbal cues and they may reason that the more cues they can rely upon, the better their detection levels. Furthermore, restricting the presentation mode of suspect statements such that nonverbal cues are excluded, e.g., by focussing on (verbatim) transcripts, takes time, but this is often an issue for police officers. Even so, our results suggest that the mere instruction to attend to verbal cues might increase lie detection accuracy in a naturalistic setting. Research has already shown that training people in how to use content cues increases their detection levels more than training them in nonverbal cues [21]. However, for the majority of the studies included in their meta-analysis, statements were presented in the form of transcripts, thereby automatically excluding nonverbal and vocal cues. Consequently, future studies should investigate whether these strong nonverbal cues can be ignored during deception detection, solely by giving the instruction to do so.

In sum, our data demonstrated that both police officers and laypersons hold many incorrect beliefs about the diagnosticity of nonverbal cues, but were less inclined to overestimate the relationship between verbal cues and deception. Here, beliefs fitted better with what we know from research. Although various studies have already shown the dangers of relying on stereotypical non-verbal cues, the current study revealed people still believe these cues to be helpful when unmasking liars. For practitioners these stereotypical beliefs are potentially harmful (e.g., lie bias), therefore, their diagnosticity–or the lack thereof–should, at the very least, be discussed during police training. Officers should be confronted with these mistaken beliefs and informed about more diagnostic cues. Becoming aware of these wrongful beliefs might be enough to shift their attention to verbal cues, about which—according to our findings—beliefs should be more accurate. This study further investigated whether SCAN’s intuitively appealing items might explain the popularity of the method, but results indicated no strong endorsement of SCAN items.

## Appendix A

(order as presented to all participants)

1. Denial of allegation–SCAN
2. Social introduction–SCAN
3. Coherence—CBCA
4. Clarity–RM
5. Spontaneous corrections–CBCA and SCAN
6. Lack of memory–CBCA and SCAN
7. Contradictions–CBCA
8. Perceptual information–RM
9. Main event of the statement–SCAN
10. Emotions–CBCA, RM and SCAN
11. Quantity of details–CBCA
12. Spatial information–RM and CBCA
13. Objective versus subjective time–SCAN
14. Unstructured production–CBCA
15. Description of interaction–CBCA
16. Temporal information–RM and CBCA
17. Self- references
18. Extraneous information–SCAN and CBCA
19. Missing information–SCAN
20. Reproduction of conversation–CBCA
21. Reconstructability–RM
22. First person singular, past tense–SCAN
23. Use of pronouns–SCAN
24. Unusual details–CBCA
25. Plausibility–RM
26. Changes in language–SCAN
27. Length of the statement
28. Cognitive operations–RM
